# Preoperative Low-Density Lipoprotein Apheresis for Preventing Recurrence of Focal Segmental Glomerulosclerosis after Kidney Transplantation

**DOI:** 10.1155/2018/8926786

**Published:** 2018-04-02

**Authors:** Akihito Sannomiya, Toru Murakami, Ichiro Koyama, Kosaku Nitta, Ichiro Nakajima, Shohei Fuchinoue

**Affiliations:** ^1^Department of Surgery, Kidney Center, Tokyo Women's Medical University, Tokyo, Japan; ^2^Department of Medicine, Kidney Center, Tokyo Women's Medical University, Tokyo, Japan

## Abstract

**Background:**

Focal segmental glomerulosclerosis (FSGS) often develops rapidly and frequently progresses to renal failure, while the recurrence rate after kidney transplantation is 20–50%. We performed low-density lipoprotein (LDL) apheresis before kidney transplantation in FSGS patients to prevent recurrence.

**Methods:**

Five adult patients with chronic renal failure due to FSGS undergoing living related donor kidney transplantation were investigated retrospectively. LDL apheresis was done 1-2 times before transplantation. Postoperative renal function and recurrence of FSGS were assessed.

**Results:**

The patients were two men and three women aged 24 to 41 years. The observation period ranged from 60 days to 22 months. Preoperative LDL apheresis was performed once in one patient and twice in four patients. Blood LDL cholesterol levels were normal before LDL apheresis and remained normal both after LDL apheresis and after kidney transplantation. Additional LDL apheresis was performed once in one patient with mild proteinuria after transplantation. The renal graft survived in all patients and there was no evidence of recurrent FSGS.

**Conclusions:**

Although the observation period was short, FSGS did not recur in all 5 patients receiving preoperative LDL apheresis. These results suggest that LDL apheresis can be effective in preventing recurrence of FSGS after kidney transplantation.

## 1. Introduction

Like minimal change nephrotic syndrome (MCNS), focal segmental glomerulosclerosis (FSGS) often develops rapidly with severe proteinuria and edema. However, FSGS is often refractory or intractable with a high risk of progression to renal failure, unlike MCNS, and it has been reported that the recurrence rate of FSGS after kidney transplantation is 20–50% [1–6]. To treat recurrent FSGS after kidney transplantation, steroid pulse therapy, plasma exchange, and immunosuppressive agents are employed, and administration of rituximab (an anti-CD20 monoclonal antibody) has also been tried recently, with a number of reports stating that plasma exchange and rituximab are effective [7–10]. There have also been reports that low-density lipoprotein (LDL) apheresis is effective for recurrent FSGS after kidney transplantation [11, 12]. We postulated that performing LDL apheresis before kidney transplantation might prevent recurrent FSGS. Accordingly, we performed LDL apheresis before kidney transplantation in patients with FSGS to prevent recurrence after transplantation and good results were obtained. To our knowledge, this is the first report about prophylactic LDL apheresis before kidney transplantation to prevent recurrence of FSGS.

## 2. Patients and Methods

**Figure 1:**
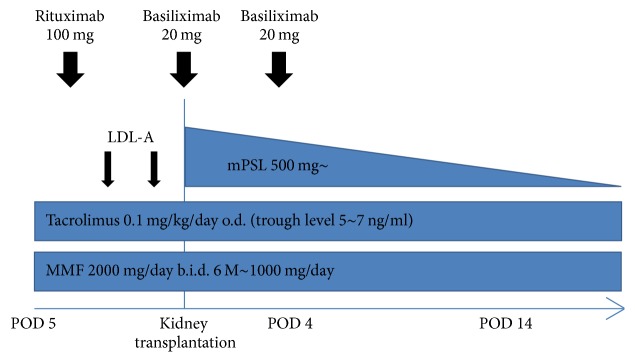
Immunosuppressive therapy for kidney transplantation and LDL apheresis to prevent recurrence of FSGS. LDL-A, low-density lipoprotein apheresis; mPSL, methylprednisolone; MMF, mycophenolate mofetil.

This study is a retrospective, observational, single-center cohort study. The study protocol was approved by the Institutional Review Boards at Tokyo Women's Medical University Hospital, Tokyo. Consecutive five adult patients with FSGS who underwent living related donor kidney transplantation were retrospectively registered as subjects in this study from July 1, 2015, to March 31, 2017. Each patient was informed about the outline of written informed consent before registration in the study. This study was conducted in accordance with Declaration of Helsinki. Clinical characteristics of the patients are described in Table 1. All cases were proved to be FSGS by renal biopsy. Their mean age at the onset of FSGS was 25.2 years (range: 11–40 years) and mean age at transplantation was 32.6 years (range: 24–41 years). The donor was the mother in all five patients. Three patients were not on dialysis before transplantation, while the preoperative duration of dialysis was two months and 29 months in the remaining two patients. The three nondialysis patients received preemptive kidney transplantation. All three cases of preemptive transplantation presented nephrotic syndrome with proteinuria more than 3.5 g per day and hypoalbuminemia less than 3.0 g/dL before transplantation. Preoperatively, anti-HLA donor antibody was negative in all five patients. The immunosuppressive protocol for living donor kidney transplantation was as follows. At four days before transplantation, rituximab (100 mg) was administered intravenously, and immunosuppressive therapy was initiated with methylprednisolone, tacrolimus, and mycophenolate mofetil. Basiliximab (anti-CD25 antibody) was administered intravenously at a dose of 20 mg during transplantation and at four days after transplantation. In Case 1, plasma exchange was performed twice before transplantation to prevent rejection by reducing anti-blood group antibody titers for ABO-incompatible transplantation, and LDL apheresis was performed once at one day before transplantation. In Cases 2–5, LDL apheresis was performed twice at three days and one day before transplantation without plasma exchange (Figure 1). All five cases did not have experience of LDL apheresis before. LDL apheresis was performed with a dextran sulfate column, with 3000–4000 mL of plasma being treated over 2-3 hours. The blood level of LDL cholesterol, serum creatinine, estimated glomerular filtration rate (eGFR), and urine protein excretion were monitored over time, with the postoperative observation period ranging from 2 months to 22 months.

## 3. Results

Perioperative clinical parameters are described in Table 2. LDL cholesterol was measured before LDL apheresis and was within the normal range in all patients. In all five patients, the LDL cholesterol level measured immediately after LDL apheresis and the most recent LDL level during follow-up were also within the normal range. The lipid abnormality was not tried to be ameliorated by statin or any medication in all cases. Proteinuria disappeared until two weeks after transplantation without additional treatment in all five cases. In Case 2, urinary protein increased (qualitative test: from ± to 2+) at five months after transplantation, so one additional LDL apheresis session was performed, after which urinary protein remained normal. The mean serum creatinine level was 0.111 mmol/L (range: 0.07–0.152 mmol/L) at one month after transplantation, while the most recent mean serum creatinine was 0.111 mmol/L (range: 0.076–0.143 mmol/L). eGFR ranged from 38.2 mL/min/1.73 m^2^ to 90.8 mL/min/1.73 m^2^ at one month after transplantation, while the latest eGFR values ranged from 31.7 mL/min/1.73 m^2^ to 83.4 mL/min/1.73 m^2^. Qualitative tests for urinary protein ranged from − to 2+ throughout the follow-up period. In three patients, the quantitative urinary protein-to-creatinine ratio ranged from 0.17 g/gCr to 0.87 g/gCr at one month after transplantation, while the latest values ranged from 0.04 g/gCr to 0.88 g/gCr. The graft survived with no rejection reactions in any of the patients. In addition, there were no signs or symptoms suggesting recurrence of FSGS in any of the patients. Biopsy of the transplanted kidney was not performed in any patient.

## 4. Discussion

We suggested that performing LDL apheresis before kidney transplantation in patients with FSGS can prevent the recurrence and good results were obtained.

Persistent severe albuminuria reduces the serum albumin level and leads to a compensatory increase of albumin synthesis in the liver, but lipoprotein synthesis is also increased simultaneously and hyperlipidemia occurs. In addition, the enzyme catabolizing lipoproteins is excreted in the urine; this exacerbates hyperlipidemia. The resulting increased uptake of oxidized LDL by glomerular mesangial cells causes mesangial hyperplasia and leads to glomerular sclerosis [13]. Furthermore, uptake of lipid droplets by renal tubular cells causes tubulointerstitial damage. Moreover, phagocytosis of lipids by macrophages in the blood vessel walls leads to progression of arteriosclerosis, which also worsens renal damage. Thus, elevated lipid levels accelerate renal dysfunction by affecting the renal tubules and blood vessels in addition to the glomeruli.

LDL apheresis is performed to reduce lipid levels. It has been reported that improvement of hyperlipidemia protects the renal blood vessels, decreases oxidative stress, and suppresses induction of macrophages [14, 15]. It has also been reported that LDL apheresis eliminates humoral factors. FSGS can recur after kidney transplantation, and it was suggested that a humoral factor influencing glomerular permeability may be involved in its recurrence [16]. Levels of inflammatory cytokines, such as tumor necrosis factor *α* and interleukin-8, are often increased in nephrotic syndrome including FSGS [17], and eliminating such humoral factors by LDL apheresis can decrease proteinuria. Nakamura et al. reported that LDL apheresis decreased urinary protein loss and excretion of podocytes in patients with diabetic nephropathy [18], suggesting that apheresis decreases proteinuria through a protective effect on podocytes.

LDL apheresis was first introduced for treatment of familial hyperlipidemia [19]. In 1988, Tojo et al. reported that LDL apheresis was useful for patients with nephrotic syndrome due to drug-resistant FSGS [20]. Subsequently, the Kansai FGS LDL Apheresis Treatment (K-FLAT) Study Group showed that LDL apheresis was effective for refractory nephrotic syndrome [21]. Additionally, a retrospective study showed that the improvement rate of symptoms was 62% at two years after LDL apheresis and increased to 86% at five years after LDL apheresis [22]. The results of POLARIS (Prospective Observational Survey of the Long-Term Effect of LDL-Apheresis on Drug-Resistant Nephrotic Syndrome), a prospective cohort study of LDL apheresis for drug-resistant and refractory nephrotic syndrome, were reported recently [23, 24]. The subjects included patients with FSGS, MCNS, membranous nephropathy, renal amyloidosis, lupus nephritis, membranoproliferative glomerulonephritis, and crescentic glomerulonephritis. After prospective observation for two years, proteinuria decreased to 1 g/day or less in 47.7% of the patients, indicating the usefulness of LDL apheresis.

In patients with FSGS, recurrence of this disease after kidney transplantation can adversely influence the function of the transplanted kidney, so effective treatment of recurrence is important [25, 26]. The recurrence rate of FSGS is 20–50% after transplantation and recurrence eventually leads to graft loss which occurs in about half of the patients [27]. Yanagisawa et al. performed LDL apheresis in four patients with nephrotic syndrome after kidney transplantation [11]. Kidney biopsy revealed chronic rejection in all four patients and FSGS in two of them, while LDL apheresis rapidly decreased urinary protein loss in all four. According to Ideura et al. [28], LDL apheresis and administration of simvastatin were effective in patients with membranous nephropathy after kidney transplantation. Furthermore, Masutani et al. [12] reported that LDL apheresis decreased urinary protein loss in patients with recurrence of FSGS after kidney transplantation although their recurrent FSGS showed resistance to plasma exchange therapy. Thus, several authors have used LDL apheresis to treat recurrent FSGS after kidney transplantation, but there have been no reports on performance of LDL apheresis before transplantation to prevent recurrence of this condition.

Couser [29] reported that the mean time to recurrence of FSGS is short, 10–14 days. Ponticelli and Glassock [30] reported that two patterns of clinical presentations of recurrent FSGS after transplantation are recognized: (1) an early recurrence characterized by a massive proteinuria within hours to days after transplantation and (2) a late recurrence that develops insidiously several months or years after transplantation. At least super rapid recurrence of FSGS such as 1–14 days after transplantation could be prevented in the present five cases.

Iguchi et al. [31] reported that the beneficial effect of pretreatment by plasma exchange was suggested. The possibility of decreasing or absorbing the potent pathogenic permeability factor might be expected in both LDL apheresis and plasma exchange. We considered that LDL apheresis has advantage without using blood products.

Korbet [32] examined the correlation between proteinuria and outcomes in clinical studies of FSGS and reported that there were major differences between nephrotic and nonnephrotic patients. Therefore, when patients with FSGS undergo kidney transplantation, preventing postoperative recurrence is extremely important, and our present findings suggest that performing LDL apheresis before kidney transplantation can prevent the recurrence of FSGS.

This study's limitation is that the sample size consists only of five patients; further research with a larger sample is needed.

## 5. Conclusion

We performed LDL apheresis in five patients before kidney transplantation to prevent recurrence of FSGS. Although the observation period was short, no recurrence was observed. It has already been reported that LDL apheresis is effective in treatment of FSGS and for recurrent FSGS after kidney transplantation. Our investigation suggested that performing LDL apheresis before transplantation may have a prophylactic effect against recurrence of FSGS. However, long-term evaluation of the outcome in a larger study is required to confirm these findings.

## Figures and Tables

**Table 1:** Clinical characteristics of the patients.

	Case 1	Case 2	Case 3	Case 4	Case 5
Age at kidney transplantation (years)	41	24	38	33	27
Age at onset of FSGS (years)	40	11	32	26	17
Gender (male/female)	Female	Male	Female	Male	Female
Donor relationship	Mother	Mother	Mother	Mother	Mother
Dialysis duration (months)	2	29	0	0	0
Preoperative anti-HLA donor antibody	Negative	Negative	Negative	Negative	Negative
Preoperative dose of rituximab (mg)	100	100	100	100	100
Preoperative PE sessions (number)	2	0	0	0	0
Preoperative LDL apheresis sessions (number)	1	2	2	2	2
Postoperative observation period (months)	22	19	14	3	2

FSGS, focal segmental glomerulosclerosis; HLA, human leukocyte antigen; PE, plasma exchange; LDL, low-density lipoprotein.

**Table 2:** Perioperative clinical parameters.

	Case 1	Case 2	Case 3	Case 4	Case 5
Preoperative LDL-A LDL cholesterol (mmol/L)	3.54	2.20	3.57	1.99	1.78
Postoperative LDL-A LDL cholesterol (mmol/L)	2.74	0.54	2.15	1.50	1.27
Latest LDL cholesterol (mmol/L)	2.30	2.97	2.56	1.58	1.37
Postoperative LDL apheresis (number)	0	1	0	0	0
Serum creatinine at one month after transplantation (mmol/L)	0.103	0.070	0.114	0.152	0.114
Latest serum creatinine level (mmol/L)	0.122	0.076	0.104	0.143	0.111
eGFR at one month after transplantation (mL/min/1.73 m^2^)	42	90.8	38.2	39.3	42.1
Latest eGFR (mL/min/1.73 m^2^)	31.7	83.4	41.5	42.0	43.2
Qualitative urinary protein at one month after transplantation	−	2+	−	1+	1+
Latest qualitative urinary protein	2+	−	−	1+	1+
Quantitative urinary protein at one month after transplantation (urine protein/creatinine ratio)	0.17	NA	NA	0.30	0.87
Latest quantitative urinary protein (urine protein/creatinine ratio)	0.88	0.18	0.04	0.27	0.55

LDL, low-density lipoprotein; eGFR, estimated glomerular filtration rate; NA, not available.
